# The Effectiveness of Plaza Dancing on Psychological Well-Being and Ill-Being: A Systematic Review and Meta-Analysis

**DOI:** 10.3389/fpsyg.2022.864327

**Published:** 2022-04-15

**Authors:** Zhenggang Bai, Yixuan Li, Yufan Yang, Chengdan Xie, Zhengyun Zhu, Yan Xu, Ruhai Bai

**Affiliations:** ^1^Evidence-Based Research Center of Social Science & Health, Nanjing University of Science & Technology School of Public Affairs, Nanjing, China; ^2^Gansu Province Center for Disease Control and Prevention, Lanzhou, China

**Keywords:** square fitness dance, mental health, psychological health, mental hygiene, plaza dancing

## Abstract

Evidence suggests that participation in plaza dancing may affect mental health. This study for the first time quantified the relationships between plaza dancing and psychological well-being and ill-being. We systematically searched PubMed, Web of Science, CNKI, Wanfang, and VIP to identify relevant studies published from the databases since their inception to July 25, 2021. The standardized mean differences (SMDs) of pre-to-post intervention data were calculated in the meta-analysis. Subgroup and meta-regression analyses were performed to test the potential moderating effects of age, outcome classification, measurement instruments, district, publication year, total sample size, and the duration, frequency, and length of the square dance intervention. A total of 25 original articles met all the eligibility criteria and were included in the review, and 17 studies were included in the meta-analysis. The meta-analysis revealed that plaza dancing improved psychological well-being (pooled SMD = 0.76; 95% CI: 0.58, 0.95; *I*^2^ = 86.9%) and reduced psychological ill-being (pooled SMD = −0.84; 95% CI: −1.00, −0.68; *I*^2^ = 64.8%). The study participants’ age and district did not seem to affect the effectiveness of the plaza dancing intervention. The duration and frequency of plaza dancing affected the association between square dance and psychological well-being (duration, β = −0.044; 95% CI: −0.085, −0.004; frequency, β = 0.122; 95% CI: 0.024, 0.221) and psychological ill-being (duration, β = −0.029; 95% CI: −0.040, −0.018; frequency, β = 0.154; 95% CI: 0.030, 0.278). Plaza dancing has a significant positive effect on psychological well-being and psychological ill-being, and the effects are moderated by intervention modality. Generalizing plaza dancing interventions to promote psychological well-being and prevent or treat psychological ill-being is needed.

**Systematic Review Registration:**

[www.crd.york.ac.uk/prospero], identifier [CRD42021272016].

## Introduction

Psychological ill-being is a disorder of mental activity that leads to abnormalities in cognition, emotion, and behavior. Thus, the affected individuals cannot maintain a normal mental life and may act in ways that endanger themselves and the community, and psychological ill-being accounts for a substantial proportion of the disease burden worldwide (Prince et al., 2007; Biddle and Asare, 2011; Whiteford et al., 2013). Individuals with psychological ill-being may experience low self-esteem, struggle to maintain interpersonal relationships, and have a higher risk of communicable and non-communicable diseases than those not experiencing psychological ill-being (WHO, 2001; Prince et al., 2007). Psychological well-being, however, is a positive state of being that allows the individuals to realize their potential, experience positive emotions, cope with stress, maintain interpersonal relationships, work productively, and contribute to their community (Herrman et al., 2005). The diagnosis of mental disorders depends not only on the absence of psychological well-being but also on the presence of psychological ill-being (Rodriguez-Ayllon et al., 2019).

For the purpose of this review, the term psychological ill-being will be used to represent unpleasant feelings or emotions that impact the level of functioning as well as preclinical and clinically diagnosed psychological disorders (e.g., negative affect, depression, anxiety, hostility, anger, etc.) (Antaramian et al., 2010). Conversely, psychological well-being is the combination of positive affective states and the ability to function with optimal effectiveness in personal and social life (e.g., life satisfaction, energy, happiness, positive affect, and self-esteem) (Antaramian et al., 2010).

Plaza dancing is an increasingly popular form of exercise in China. It is also known as square fitness dance and refers to a unified dance spontaneously performed by people in squares, parks, streets, lanes, and open spaces in front of buildings, accompanied by music (Gao et al., 2016). The activity is carried out nationwide and is especially favored by middle-aged and older people (Wang, 2014). Compared to traditional exercises, such as running and swimming, plaza dancing is characterized by simple movements that can be easily learned by beginners. Additionally, it is not restricted by time, theme, or rhythm. Compared with indoor or individual exercise (e.g., tennis, yoga, etc.), such kind of outdoor group-based and music-guided exercise may show superior impacts in promoting mental well-being (Gao et al., 2016).

Some recent original studies found that plaza dancing could effectively improve the somatization, depression, and anxiety symptoms of middle-aged and older women (Yang et al., 2014). Research by Zhou and Li (2014) found that plaza dancing is more suitable for long-term practice and has a tendency to be better than walking in terms of psychological and emotional aspects. Another study pointed out that plaza dancing can relieve the negative psychological symptoms of team members, such as utilitarian thoughts, nervousness, and anxiety (Bai, 2004). Some original studies focused on the characteristics of dance interventions in terms of duration, frequency, length, or classification, which suggests that dance benefits mental health (Biddle and Asare, 2011; Chekroud et al., 2018). Moreover, previous reviews have reported evidence concerning the effects of dance intervention on fitness and mental health (Predovan et al., 2018; Barnish and Barran, 2020; Wu et al., 2021). However, these reviews focused on the impact of dance intervention on mental health, and no systematic review or meta-analysis paid attention to the relationship between plaza dancing and psychological well-being and ill-being.

Therefore, this systematic review and meta-analysis investigated the effects of plaza dancing on psychological well-being and psychological ill-being. This study is timely and critical to designing prevention and treatment strategies for mental health conditions, further helping to improve mental health globally.

## Methods

This study was performed following the Preferred Reporting Items for Systematic Reviews and Meta-Analyses (PRISMA) (Moher et al., 2009) and the Cochrane Collaboration Handbook (Higgins and Green, 2011) guidelines. The protocol was registered on the International Prospective Register of Systematic Reviews (PROSPERO, CRD42021272016).

### Literature Search and Study Selection

A systematic literature search was performed in five electronic bibliographic databases: PubMed, Web of Science, CNKI, Wanfang, and VIP. The dates of the published articles included in the search were from the earliest records to July 25, 2021. We developed a search strategy for the databases based on keywords of relevant articles that we previously identified. The search strategy included all combinations of terms related to plaza dancing, psychological well-being, and psychological ill-being. A summary of the search strategy is provided in the Supplementary Text.

The reference lists of all the eligible articles were reviewed to find other literature that had been missed in the initial searches. Articles identified from the reference lists were further screened and evaluated using the same criteria. Reference searches were repeated on all newly identified articles until no additional relevant articles were found.

### Study Selection Criteria

Studies were included if they (a) reported plaza dancing used in interventions and that the plaza dancing took place in any location, including squares, parks, streets, and open spaces in front of buildings (intervention); (b) included a comparison group with no plaza dancing intervention (i.e., education, walking, or no physical activity) (comparison); (c) reported the association between plaza dancing and at least one type of psychological ill-being (i.e., depression, anxiety, nervousness, or negative affect) and/or psychological well-being (i.e., self-esteem, self-image, positive affect, energy, happiness, and life satisfaction) as the outcome (outcome); and (d) were randomized controlled trials (RCTs), cluster-randomized trials, or quasi-RCTs, or had an intervention study design involving a control group (study design).

Studies were excluded if they (a) were only published as abstracts, comments, or reviews; (b) reported that plaza dancing was combined with other types of interventions (e.g., co-interventions, such as a dietary program or physical activity combined with plaza dancing), because they preclude drawing conclusions on the isolated effect of plaza dancing on the psychological well-being and ill-being outcomes; and (c) reported that participants had physical disabilities and/or targeted purely clinical populations (e.g., individuals who took medicine as directed) (population).

### Data Extraction and Preparation

A standardized data extraction form was developed to collect the study information, including (a) study-level data (country, author, publication year, description of plaza dancing, and psychological well-being and ill-being outcomes), (b) sample-level data (participants’ age and gender), and (c) effect sizes. The data were extracted independently by two reviewers, and disagreements were reviewed and resolved through discussion.

According to the Cochrane Collaboration Handbook (Higgins and Green, 2011), the mean and standard deviation (SD) values of the pre-to-post intervention differences were first extracted. More specifically, the change values, if not reported, were calculated by “Mean _change_ = Mean_post_ − Mean_pre_” and “SD_change_ = SQRT[(SD_pre_^2^ + SD_post_^2^) − (2*Corr*SD_post_ *SD_pre_)]”, where the correlation coefficient (Corr) was set as 0.5. For those studies that only provided “SE” and “95% CI”, the SD was calculated based on the following formula: (1) SD = SE* SQRT (N), where N is the sample size; (2) SD = SQRT (N) * [(UCI − LCI)/3.92], where U = upper CI and L = lower CI.

### Study Quality Assessment

Two authors independently assessed the quality of the articles using the United States National Institutes of Health (NIH)’s Quality Assessment Tool for Controlled Intervention Studies^1^. These assessment tools rate each study based on 14 criteria. For each criterion, a score of one was assigned if “yes” was the response, whereas a score of zero was assigned otherwise (i.e., an answer of “no,” “not applicable,” “not reported,” or “cannot determine”). A study-specific global score ranging from 0 to 14 was calculated by summing the scores across all criteria. This assessment measured the strength of scientific evidence but was not used to determine the inclusion of studies. The assessment results are presented in Supplementary Table 1. In general, the studies were of reasonably high quality, with a mean quality score of 8.56.

### Statistical Analysis

A meta-analysis was performed based on the findings obtained from 17 of the 25 included studies to estimate the pooled effects of plaza dancing on psychological well-being and psychological ill-being. Only those studies that provided the needed information for pre-to-post intervention measurements and had a control group were included in the meta-analysis. The meta-analysis was performed for two specific conditions: (1) psychological well-being and (2) psychological ill-being. If studies reported multiple results in each article, the results of psychological well-being and psychological ill-being were all included.

Considering the different outcomes used in the studies, standardized mean differences (SMDs) of pre-to-post intervention measures were calculated and given weight by their inverse variance. Study heterogeneity was assessed using the *I*^2^ index and tau-squared (*T*^2^) statistics. The level of heterogeneity represented by *I*^2^ was interpreted as modest (*I*^2^ ≤ 25%), moderate (25% < *I*^2^ ≤ 50%), substantial (50% < *I*^2^ ≤ 75%), or considerable (*I*^2^ > 75%) (Barberio et al., 2021). In our meta-analysis, a fixed-effects model was used when modest to moderate heterogeneity was present, and a random-effects model was used when substantial to considerable heterogeneity was present (Barberio et al., 2021).

Subgroup and meta-regression analyses were used to test the potential moderating effects of age, outcome classification, measurement instruments, country, publication year, total sample size, and the duration, frequency, and length of the plaza dancing intervention. A prespecified sensitivity analysis was conducted to investigate the influence of a single study on the overall pool estimation by omitting one study at a time. Publication bias was assessed by the visual inspection of the funnel plots and Begg’s and Egger’s tests.

All the statistical analyses were conducted in STATA with specific commands (e.g., metan and metareg) (version 14.0; Stata Corp., College Station, TX, United States). All the analyses used two-sided tests, and *P* < 0.05 was considered to indicate statistical significance.

## Results

### Literature Search and Characteristics of the Included Studies

**FIGURE 1:**
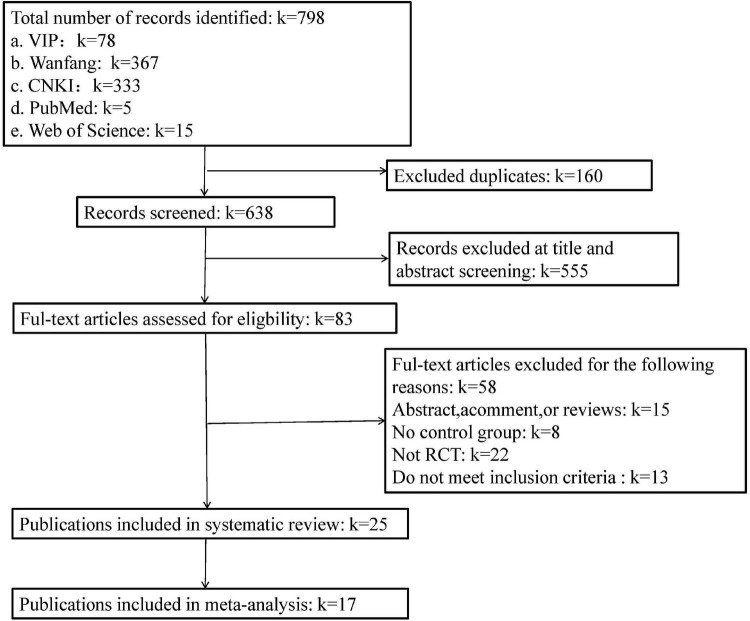
A flowchart of the literature search and study selection according to the PRISMA standard.

Our search yielded 798 articles (Figure 1). After removing 160 duplicates, 638 titles and abstracts were screened. We excluded 555 records based on the screening of titles and abstracts. Full texts of the remaining 83 articles were reviewed against the selection criteria. In total, 25 articles (Wang, 2014, 2016, 2017; Zhang et al., 2014; Chen and Jia, 2015; Jia, 2015; Lv, 2015; Gao et al., 2016; Han, 2016; Li, 2016, 2017; Shen, 2016; Guan, 2017; Guo, 2017; Guan et al., 2017; Hao, 2017; Pang, 2017; Lu, 2019; Sun, 2019; Wu and Li, 2019; Sun and Wang, 2020; Wang et al., 2020; Zeng et al., 2020; Wang et al., 2021; Yang et al., 2021) that reported the associations between plaza dancing and psychological well-being or/and psychological ill-being were included in this review. Of them, 17 were included in the meta-analysis (Zhang et al., 2014; Gao et al., 2016; Han, 2016; Li, 2016, 2017; Shen, 2016; Wang, 2016, 2017; Guo, 2017; Guan et al., 2017; Pang, 2017; Sun, 2019; Wu and Li, 2019; Wang et al., 2020; Zeng et al., 2020; Wang et al., 2021; Yang et al., 2021).

All these studies were conducted in China and were published between 2014 and 2021. The studies varied in sample size, ranging from 20 to 579. Two studies recruited young subjects aged 18–21 years, 21 studies recruited subjects aged 30–81 years, and two studies did not report the mean age (or age range). Thirteen studies recruited women, 10 studies recruited men and women, and one study did not report the number of women. The duration of plaza dancing ranged broadly from 8 weeks to 18 months, the frequency ranged from 3 to 7 times/week, and the length ranged from 40 to 120 min/time. Ten studies included a control group that performed other types of physical activity (i.e., walking exercise, table tennis, and belly dance), eight studies included a control group that maintained their usual lifestyle, and seven studies had no intervention.

Ten studies provided results on psychological well-being (including life satisfaction, energy, happiness, positive affect, and self-esteem), six studies provided results on psychological ill-being (including negative affect, depression, anxiety, hostility, and anger), and nine studies provided results on both. Psychological well-being and ill-being were measured using the PSPP, GWB, POMS, SCL-90, SF-36, and SAS. All studies reported that plaza dancing had a positive impact on psychological well-being and ill-being, thus realizing an improvement in the quality of life. Eight studies included only in the narrative synthesis also indicated that plaza dancing could improve psychological well-being and ill-being (Table 1).

**TABLE 1:** Main characteristics and findings of the association between plaza dancing and psychological well-being or/and ill-being.

First author (publication year)	Country	Sample size (total (female)		Age (mean ± *SD*/range)		Plaza dancing group			Control group	Outcomes		Effectiveness
		Plaza dancing	Control group	Plaza dancing	Control group	Duration	Frequency	Length		Instruments	Classification	
(1) Wang et al., 2021	Jiaozuo, Henan Province, China	45 (45)	46 (46)	56.92 ± 7.17	58.17 ± 6.30	12 weeks	5 times/week	60 min/time	Walking exercise	LSIA; PEES	(1) Psychological well-being: life satisfaction; (2) Psychological ill-being: negative affect	(+)
(2) Yang et al., 2021	Chongqing, China	20 (20)	20 (20)	–	–	12 weeks	3 times/week	87 min/time	No intervention	GWB	Psychological well-being: energy, life satisfaction, happiness, positive affect, and relaxation	(+)
(3) Sun and Wang, 2020	Zhenjiang, Jiangsu Province, China	40 (28)	40 (22)	60.87 ± 5.42	61.03 ± 5.38	3 months	≥3 times/week	≥1 h/time	Walking exercise	BFS	(1) Psychological well-being: happiness, vitality, and calm; (2) Psychological ill-being: depression, thinking, anger, excitement, and no vitality	(+)
(4) Zeng et al., 2020	Chengdu, Sichuan Province, China	30 (30)	30 (30)	63.20 ± 4.27	63.20 ± 4.29	3 months	5–7 times/week	60–90 min/time	Maintained their usual lifestyle	SCL-90; POMS	(1) Psychological well-being: energy and self-esteem; (2) Psychological ill-being: somatization, compulsive symptoms, interpersonal sensitivity, depression, anxiety, hostility, terror, paranoid, psychosis, nervousness, anger, fatigue, and panic, TMD	(+)
(5) Lu, 2019	Dalian, Liaoning Province, China	294 (276)	285 (–)	55–75	55–75	5 months	≥3 times/week	≥1 h/time	Participate in other physical exercises	PSPP; GWB	Psychological well-being: energy, life satisfaction, happiness, positive affect, relaxation, and self-image	(+)
(6) Sun, 2019	Hohhot, Inner Mongolia, China	106 (106)	111 (111)	45–55	45–55	8 weeks	≥3 times/week	≥40 min/time	No intervention	GWB; PSPP	Psychological well-being: energy, life satisfaction, happiness, positive affect, relaxation, general well-being, and self-image	(+)
(7) Wang et al., 2014	Changchun, Jilin Province, China	33 (26)	33 (21)	81.06 ± 5.17	81.09 ± 7.44	12 weeks	3 times/week	40 min/time	Maintained their usual lifestyle	GDS-15; SF-36	(1) Psychological well-being: quality of life; (2) Psychological ill-being: depression	(+)
(8) Wu and Li, 2019	Luoyang, Henan Province, China	25 (25)	25 (25)	–	–	2 months	4 times/week	76 min/time	Maintained their usual lifestyle	GWB	Psychological well-being: energy, life satisfaction, happiness, positive affect, and relaxation	(+)
(9) Guo, 2017	Beijing, China	16 (16)	16 (16)	55.10 ± 3.44	55.80 ± 3.92	12 weeks	3 times/week	40 min/time	No intervention	BDI; SES	(1) Psychological well-being: self-esteem; (2) Psychological ill-being: depression	(+)
(10) Guan, 2017	Xinxiang, Henan Province, China	16 (–)	16 (–)	60–70	60–70	3 months	3–4 times/week	≥50 min/time	Maintained their usual lifestyle	SCL-90	Psychological ill-being: somatization, compulsive symptoms, interpersonal sensitivity, depression, anxiety, hostility, terror, paranoid, and psychosis	(+)
(11) Guan, 2017	Changsha, Hunan Province, China	50 (47)	50 (43)	64.30 ± 5.60	65.40 ± 6.90	3 months	≥3 times/week	≥1 h/time	Only conduct daily basic activities	SAS; SDS	Psychological ill-being: anxiety and depression	(+)
(12) Hao, 2017	Shanghai, China	25 (25)	25 (25)	59.00	59.00	24 weeks	3 times/week	90 min/time	Maintained their usual lifestyle	PESF; PEDQ	Psychological well-being: self-cognition (self-satisfaction, self-image, physical condition satisfaction), personal emotions (feeling happiness, positive affect, feeling confident, cheerful), and interpersonal relationships (willing to communicate)	(+)
(13) Li, 2017	Pingdingshan, Henan Province, China	30 (15)	30 (15)	61.80	62.60	3 months	3 times/week	≥40 min/time	Participate in running, walking, and cycling	PSPP; GWB	Psychological well-being: energy, life satisfaction, happiness, positive affect, relaxation, and self-image	(+)
(14) Pang, 2017	Jinan, Shandong Province, China	20 (20)	20 (20)	30–50	30–50	2 months	4 times/week	71 min/time	No intervention	PSPP; GWB	Psychological well-being: energy, life satisfaction, happiness, positive affect, relaxation, and self-image	(+)
(15) Wang, 2017	Qufu, Shandong Province, China	30 (30)	30 (30)	45–59	45–59	12 weeks	3 times/week	90 min/time	Walking exercise	GWB	Psychological well-being: energy, life satisfaction, happiness, positive affect, relaxation, and general well-being	(+)
(16) Gao et al., 2016	Harbin, Heilongjiang Province, China	26 (26)	24 (24)	54.50 ± 4.50	53.50 ± 4.70	3 months	≥5 times/week	60–90 min/time	No intervention	SDS	Psychological ill-being: depression	(+)
(17) Han, 2016	Puyang, Henan Province, China	25 (25)	25 (25)	45–70	45–70	8 weeks	4 times/week	45–60 min/time	Maintained their usual lifestyle	POMS	(1) Psychological well-being: energy and self-esteem; (2) Psychological ill-being: nervousness, anger, fatigue, depression, and panic, TMD	(+)
(18) Li, 2016	Nanjing, Jiangsu Province, China	10 (10)	10 (10)	45.12 ± 3.34	44.32 ± 3.21	12 weeks	3 times/week	1 h/time	Belly dance	EIEQ; CSS	(1) Psychological well-being: energetic stimulation, calm mind and body, active involvement, and self-concept; (2) Psychological ill-being: physical exhaustion	(+)
(19) Shen, 2016	Zhengzhou, Henan Province, China	10 (9)	10 (9)	61.45 ± 1.36	61.10 ± 0.99	12 weeks	4 times/week	60 min/time	Practice in running, walking, gateball, diabolo, and table tennis	SCL-90; GWB	(1) Psychological well-being: energy, life satisfaction, happiness, positive affect, relaxation, and general well-being; (2) Psychological ill-being: somatization, compulsive symptoms, interpersonal sensitivity, depression, anxiety, hostility, terror, paranoid, and psychosis	(+)
(20) Wang, 2016	Bengbu, Anhui Province, China	86 (86)	93 (93)	65.80 ± 7.80	65.80 ± 7.80	6 months	≥4 times/week	90 min/time	Maintained their usual lifestyle	SF-36	Psychological well-being: vitality, social functioning, role-emotional, and mental health	(+)
(21) Chen and Jia, 2015	Dingxi, Gansu Province, China	160 (80)	160 (80)	18.90	18.90	1 month	In-class training	100 min/time	Public physical education classes	SCL-90	Psychological ill-being: somatization, compulsive symptoms, interpersonal sensitivity, depression, anxiety, hostility, terror, paranoid, and psychosis	(+)
(22) Jia, 2015	Dingxi, Gansu Province, China	160 (80)	160 (80)	19–21	19–21	1 month	In-class training	100 min/time	Public physical education classes	SCL-90	Psychological ill-being: somatization, compulsive symptoms, interpersonal sensitivity, depression, anxiety, hostility, terror, paranoid, and psychosis	(+)
(23) Lv, 2015	Xi’an, Shanxi Province, China	25 (25)	20 (20)	53.68 ± 6.17	53.40 ± 4.22	6 months	4 times/week	2 h/time	Maintained their usual lifestyle	SCL-90; SF-36	(1) Psychological well-being: vitality, social functioning, role-emotional, and mental health; (2) Psychological ill-being: somatization, compulsive symptoms, interpersonal sensitivity, depression, anxiety, hostility, terror, paranoid, and psychosis	(+)
(24) Wang, 2014	Nanyang, Henan Province, China	20 (10)	20 (10)	55–65	55–65	3 months	7 times/week	40 min/time	No intervention	GWB	Psychological well-being: energy, life satisfaction, happiness, positive affect, and relaxation	(+)
(25) Zhang et al., 2014	Shenyang, Liaoning Province, China	30 (16)	30 (14)	65.20 ± 4.53	64.10 ± 4.36	18 months	≥4 days/week	30–60 min/day	No intervention	HAMA; HAMD	Psychological ill-being: anxiety and depression.	(+)

*BDI, Beck Depression Inventory; BFS, Befindlichkeitsskalen; CSS, Core Self-Evaluation Scale; EIEQ, Exercise-Induced Emotion Questionnaire; GDS-15, 15-item Geriatric Depression Scale; GWB, General Well-Being Schedule; HAMA, Hamilton Anxiety Scale; HAMD, Hamilton Depression Scale; LSIA, Life Satisfaction Index A; PEDQ, Personal Emotion Dimension Questionnaire; PEES, Post-Exercise Emotional Experience Scale; PESF, Personal Emotion Self-Evaluation Form; POMS, Profile of Mood States; PSPP, Platform Sizing and Performance Program; SAS, Self-Rating Anxiety Scale; SCL-90, Symptom Check List 90; SDS, Self-Rating Depression Scale; SES, Self-Esteem Scale; SF-36, the MOS item short-from health survey; TMD, total of motional disturb.*

*(+) indicates that plaza dancing has a positive impact on psychological well-being and ill-being, thus realizing the improvement of the quality of life.*

### Meta-Analysis of the Association Between Plaza Dancing and Psychological Well-Being and/or Ill-Being

**FIGURE 2:**
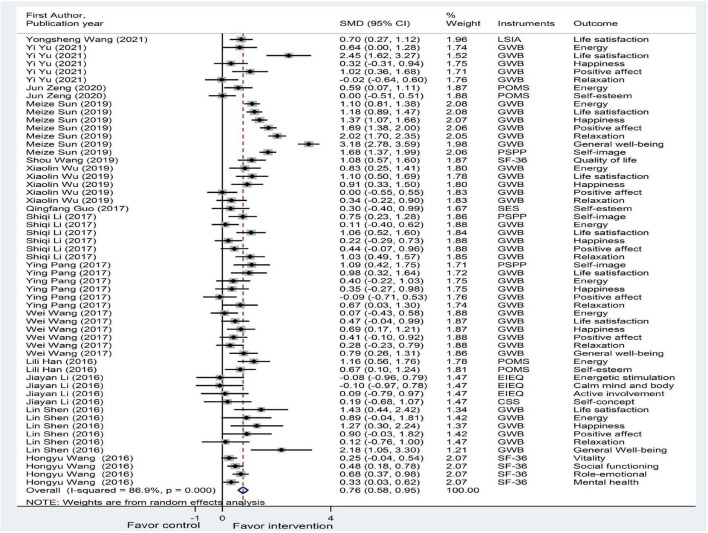
A meta-analysis of the effectiveness of plaza dancing on psychological well-being. Seven studies reported results for psychological well-being, and seven studies reported results for both psychological well-being and ill-being. The results from 14 studies on psychological well-being (reporting 56 analyses, including different related outcomes) were included. The random-effects model was used as it assumes that varying effect sizes between the studies are due to the differences in the study content and population. Positive effect size values indicate higher scores for psychological well-being in favor of the intervention. BDI, Beck Depression Inventory; CSS, Core Self-Evaluation Scale; EIEQ, Exercise-Induced Emotion Questionnaire; GDS-15, 15-item Geriatric Depression Scale; GWB, General Well-Being Schedule; HAMA, Hamilton Anxiety Scale; HAMD, Hamilton Depression Scale; LSIA, Life Satisfaction Index A; PEES, Post-Exercise Emotional Experience Scale; POMS, Profile of Mood States; PSPP, Platform Sizing and Performance Program; SAS, Self-Rating Anxiety Scale; SCL-90, Symptom Check List 90; SDS, Self-Rating Depression Scale; SES, Self-Esteem Scale; SF-36, the MOS item short-form health survey; TMD, total of motional disturb.

**FIGURE 3:**
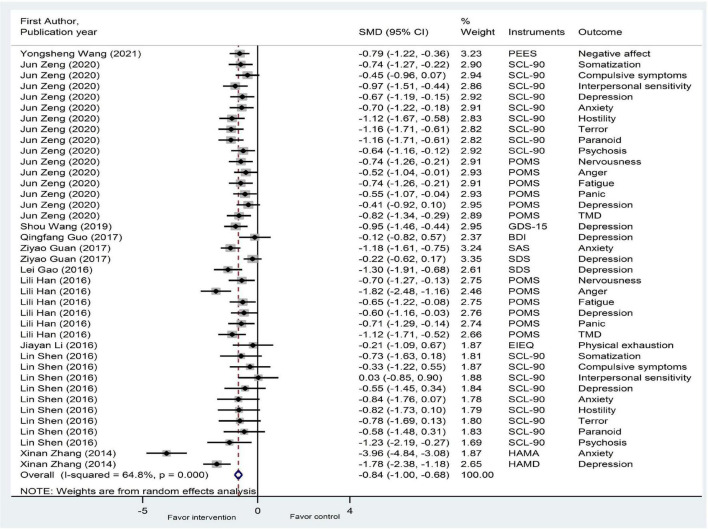
A meta-analysis of the effectiveness of plaza dancing on psychological ill-being. Three studies reported results for psychological ill-being, and seven studies reported results for both psychological well-being and ill-being. The results from 10 studies on psychological ill-being (reporting 39 analyses, including different related outcomes) were included. The random-effects model was used, as it assumes that varying effect sizes between the studies are due to the differences in the study content and population. Positive effect size values indicate lower scores for psychological ill-being in favor of the intervention. BDI, Beck Depression Inventory; CSS, Core Self-Evaluation Scale; EIEQ, Exercise-Induced Emotion Questionnaire; GDS-15, 15-item Geriatric Depression Scale; GWB, General Well-Being Schedule; HAMA, Hamilton Anxiety Scale; HAMD, Hamilton Depression Scale; LSIA, Life Satisfaction Index A; PEES, Post-Exercise Emotional Experience Scale; POMS, Profile of Mood States; PSPP, Platform Sizing and Performance Program; SAS, Self-Rating Anxiety Scale; SCL-90, Symptom Check List 90; SDS, Self-Rating Depression Scale; SES, Self-Esteem Scale; SF-36, the MOS item short-form health survey; TMD, total of motional disturb.

As shown in Figures 2, 3 and Table 2, 14 studies on psychological well-being (reporting 56 analyses, including different related outcomes) and 10 studies on psychological ill-being (reporting 39 analyses, including different related outcomes) were included in the meta-analysis. The pooled SMD of psychological well-being was 0.76 (95% CI: 0.58, 0.95), with large heterogeneity (*I*^2^ = 86.9%, *P* < 0.001; *T*^2^ = 0.407). The pooled SMD of psychological ill-being was −0.84 (95% CI: −1.00, −0.68), with substantial heterogeneity (*I*^2^ = 64.8%, *P* < 0.001; *T*^2^ = 0.165). Overall, the effect of plaza dancing on psychological well-being and psychological ill-being was statistically significant.

**TABLE 2:** Overall and subgroup meta-analyses of the effectiveness of plaza dancing on psychological well-being and ill-being.

	Sample size	% Female	Number of studies	Effect size (95% CI)	*P*-value	Heterogeneity			
						*I*^2^ (%)	χ^2^	*P*-value	Tau-squared
**(A) Psychological well-being**									
Total	4254	95.0	56	0.76 (0.58, 0.95)	<0.001	86.9	419.63	<0.001	0.407
**Outcomes classification**									
Life satisfaction	578	94.5	8	1.09 (0.76, 1.41)	<0.001	64.8	19.87	0.006	0.131
Energy	597	94.6	9	0.64 (0.34, 0.94)	<0.001	63.9	22.14	0.005	0.125
Happiness	487	93.4	7	0.73 (0.32, 1.14)	<0.001	75.0	23.97	0.001	0.216
Positive affect	487	93.4	7	0.63 (0.04, 1.23)	0.038	88.4	51.56	<0.001	0.553
Relaxation	487	93.4	7	0.66 (−0.02, 1.34)	0.058	90.9	66.02	<0.001	0.748
Self-esteem	142	100	3	0.31 (−0.10, 0.71)	0.143	31.7	2.93	0.231	0.041
General well-being	297	99.3	3	2.05 (0.31, 3.79)	0.021	96.0	50.49	<0.001	2.221
Self-image	317	90.5	3	1.21 (0.58, 1.83)	<0.001	79.8	9.88	0.007	0.240
Quality of life	66	71.2	1	1.08 (0.57, 1.60)	<0.001	–	–	–	–
Energetic stimulation	20	100	1	−0.08 (−0.96, 0.79)	0.853	–	–	–	–
Calm mind and body	20	100	1	−0.10 (−0.97, 0.78)	0.832	–	–	–	–
Active involvement	20	100	1	0.09 (−0.79, 0.97)	0.840	–	–	–	–
Self-concept	20	100	1	0.19 (−0.69, 1.07)	0.665	–	–	–	–
Vitality	179	100	1	0.25 (−0.04, 0.54)	0.096	–	–	–	–
Social functioning	179	100	1	0.48 (0.18, 0.78)	0.002	–	–	–	–
Role-emotional	179	100	1	0.68 (0.37, 0.98)	<0.001	–	–	–	–
Mental health	179	100	1	0.33 (0.03, 0.62)	0.031	–	–	–	–
**Measurement instruments**									
LSIA	91	100	1	0.70 (0.27, 1.12)	0.001	–	–	–	–
GWB	2732	94.1	38	0.86 (0.61, 1.10)	<0.001	88.1	311.37	<0.001	0.503
POMS	220	100	4	0.59 (0.12, 1.05)	0.014	65.5	8.71	0.033	0.148
PSPP	317	90.5	3	1.21 (0.58, 1.83)	<0.001	79.8	9.88	0.007	0.240
SF-36	782	97.6	5	0.52 (0.28, 0.75)	<0.001	60.9	10.24	0.037	0.042
SES	32	100	1	0.30 (−0.40, 1.00)	0.403	–	–	–	–
EIEQ	60	100	3	−0.03 (−0.54, 0.48)	0.910	0.0	0.11	0.948	0.000
CSS	20	100	1	0.19 (−0.69, 1.07)	0.665	–	–	–	–
**District**									
Central China	921	79.2	20	0.73 (0.53, 0.92)	<0.001	48.8	37.13	0.008	0.091
Southwest China	320	100	7	0.67 (0.15, 1.19)	0.012	80.4	30.55	<0.001	0.398
North China	1551	100	8	1.59 (1.13, 2.04)	<0.001	93.2	102.52	<0.001	0.391
Northeast China	66	71.2	1	1.08 (0.57, 1.60)	<0.001	–	–	–	–
East China	1396	100	20	0.43 (0.31, 0.55)	<0.001	16.7	22.80	0.246	0.012
Age (mean years)									
≤60	1680	100	27	0.83 (0.54, 1.12)	<0.001	90.3	267.04	<0.001	0.520
>60	1382	84.7	19	0.61 (0.43, 0.79)	<0.001	57.5	42.33	0.001	0.082
**(B) Psychological ill-being**									
Total	1959	–	39	−0.84 (−1.00, −0.68)	<0.001	64.8	107.97	<0.001	0.165
**Outcomes classification**									
Negative affect	91	100	1	−0.79 (−1.22, −0.36)	<0.001	–	–	–	–
Somatization	80	–	2	−0.74 (−1.19, −0.29)	0.001	0.0	0.00	0.972	0.000
Compulsive symptoms	80	–	2	−0.42 (−0.86, 0.03)	0.065	0.0	0.05	0.827	0.000
Interpersonal sensitivity	80	–	2	−0.54 (−1.51, 0.43)	0.278	72.5	3.63	0.057	0.362
Depression	498	–	9	−0.73 (−1.07, −0.39)	<0.001	70.0	26.64	0.001	0.188
Anxiety	240	–	4	−1.64 (−2.81, −0.46)	0.006	92.7	41.38	<0.001	1.307
Hostility	80	–	2	−1.04 (−1.51, −0.57)	<0.001	0.0	0.31	0.577	0.000
Terror	80	–	2	−1.06 (−1.53, −0.59)	<0.001	0.0	0.49	0.485	0.000
Paranoid	80	–	2	−0.99 (−1.51, −0.47)	<0.001	13.4	1.16	0.282	0.022
Psychosis	80	–	2	−0.79 (−1.30, −0.28)	0.002	11.3	1.13	0.288	0.020
Nervousness	110	100	2	−0.72 (−1.10, −0.33)	<0.001	0.0	0.01	0.922	0.000
Anger	110	100	2	−1.15 (−2.43, 0.12)	0.076	89.1	9.19	0.002	0.752
Fatigue	110	100	2	−0.70 (−1.08, −0.31)	<0.001	0.0	0.05	0.831	0.000
Panic	110	100	2	−0.63 (−1.01, −0.24)	0.001	0.0	0.17	0.684	0.000
TMD	110	100	2	−0.95 (−1.34, −0.55)	<0.001	0.0	0.54	0.463	0.000
Physical exhaustion	20	100	1	−0.21 (−1.09, 0.67)	0.639	–	–	–	–
**Measurement instruments**									
PEES	91	100	1	−0.79 (−1.22, −0.36)	<0.001	–	–	–	–
SCL-90	720	–	18	−0.78 (−0.94, −0.63)	<0.001	0.0	13.47	0.704	0.000
POMS	660	100	12	−0.75 (−0.94, −0.57)	<0.001	26.2	14.91	0.187	0.028
GDS-15	66	71.2	1	−0.95 (−1.46, −0.44)	<0.001	–	–	–	–
BDI	32	100	1	−0.13 (−0.82, 0.57)	0.725	–	–	–	–
SAS	100	90.0	1	−1.18 (−1.61, −0.76)	<0.001	–	–	–	–
SDS	150	93.3	2	−0.73 (−1.78, 0.32)	0.170	88.0	8.33	0.004	0.505
EIEQ	20	100	1	−0.21 (−1.09, 0.67)	0.639	–	–	–	–
HAMA	60	50.0	1	−3.96 (−4.84, −3.08)	<0.001	–	–	–	–
HAMD	60	50.0	1	−1.78 (−2.38, −1.18)	<0.001	–	–	–	–
**District**									
Central China	771	–	18	−0.77 (−0.97, −0.57)	<0.001	39.0	27.88	0.046	0.067
Southwest China	900	100	15	−0.75 (−0.88, −0.61)	<0.001	0.0	11.50	0.647	0.000
Northeast China	236	66.5	4	−1.95 (−3.03, −0.87)	<0.001	91.4	34.97	<0.001	1.092
North China	32	100	1	−0.13 (−0.82, 0.57)	0.725	–	–	–	–
East China	20	100	1	−0.21 (−1.09, 0.67)	0.639	–	–	–	–
**Age (mean years)**									
≤60	493	100	10	−0.82 (−1.09, −0.54)	<0.001	52.5	18.94	0.026	0.101
>60	1466	–	29	−0.85 (−1.05, −0.65)	<0.001	68.5	89.02	<0.001	0.195

*BDI, Beck Depression Inventory; CSS, Core Self-Evaluation Scale; EIEQ, Exercise-Induced Emotion Questionnaire; GDS-15, 15-item Geriatric Depression Scale; GWB, General Well-Being Schedule; HAMA, Hamilton Anxiety Scale; HAMD, Hamilton Depression Scale; LSIA, Life Satisfaction Index A; PEES, Post-Exercise Emotional Experience Scale; POMS, Profile of Mood States; PSPP, Platform Sizing and Performance Program; SAS, Self-Rating Anxiety Scale; SCL-90, Symptom Check List 90; SDS, Self-Rating Depression Scale; SES, Self-Esteem Scale; SF-36, the MOS item short-from health survey; TMD, total of motional disturb.*

*Seven studies reported the results of psychological well-being, three studies reported the results of psychological ill-being, and seven studies reported the results of both psychological well-being and ill-being. Results from 14 studies (reported 56 analyses, including different related outcomes) in psychological well-being and 10 studies (reported 39 analyses, including different related outcomes) in psychological ill-being were included, respectively.*

*The random-effects model was used, as it assumes that varying effect sizes between the studies are due to differences in the study content and population.*

In the subgroup analyses, the effects of plaza dancing on psychological well-being and ill-being were significantly moderated by outcome classification, measurement instruments, mean age, and district (Table 2). Life satisfaction (SMD: 1.09; 95% CI: 0.76, 1.41), energy (SMD: 0.64; 95% CI: 0.34, 0.94), happiness (SMD: 0.73; 95% CI: 0.32, 1.14), positive affect (SMD: 0.63; 95% CI: 0.04, 1.23), general well-being (SMD: 2.05; 95% CI: 0.31, 3.79), self-image (SMD: 1.21; 95% CI: 0.58, 1.83), quality of life (SMD: 1.08; 95% CI: 0.57, 1.60), social functioning (SMD: 0.48; 95% CI: 0.18, 0.78), role-emotional functioning (SMD: 0.68; 95% CI: 0.37, 0.98), mental health (SMD: 0.33; 95% CI: 0.03, 0.62), negative affect (SMD: −0.79; 95% CI: −1.22, −0.36), somatization (SMD: −0.74; 95% CI: −1.19, −0.29), depression (SMD: −0.73; 95% CI: −1.07, −0.39), anxiety (SMD: −1.64; 95% CI: −2.81, −0.46), hostility (SMD: −1.04; 95% CI: −1.51, −0.57), terror (SMD: −1.06; 95% CI: −1.53, −0.59), paranoia (SMD: −0.99; 95% CI: −1.51, −0.47), psychosis (SMD: −0.79; 95% CI: −1.30, −0.28), nervousness (SMD: −0.72; 95% CI: −1.10, −0.33), fatigue (SMD: −0.70; 95% CI: −1.08, −0.31), panic (SMD: −0.63; 95% CI: −1.01, −0.24), and TMD (SMD: −0.95; 95% CI: −1.34, −0.55) improved after the plaza dancing interventions.

Studies using the LSIA, GWB, POMS, PSPP, SF-36, PEES, SCL-90, GDS-15, SAS, HAMA, and HAMD measurement instruments showed that plaza dancing significantly improved psychological well-being and ill-being (*P* < 0.05). Finally, the subgroup analyses found that plaza dancing improved psychological well-being and ill-being regardless of the district and age.

### Results of Meta-Regression Analysis and Sensitivity Analysis

The meta-regression analysis showed that the duration and frequency of plaza dancing affected the associations between plaza dancing and psychological well-being (duration, β = −0.044; 95% CI: −0.085, −0.004; frequency, β = 0.122; 95% CI: 0.024, 0.221) and between plaza dancing and psychological ill-being (duration, β = −0.029; 95% CI: −0.040, −0.018; frequency, β = 0.154; 95% CI: 0.030, 0.278). The total sample size contributed significantly to the heterogeneity of psychological well-being studies (*P* < 0.001) (Table 3). A sensitivity analysis showed that removing individual studies from the meta-analysis did not change the association estimate in psychological well-being; however, when one study (Zhang et al., 2014) was removed from psychological ill-being studies, the heterogeneity became moderate (*I*^2^ = 36.7%, *P* = 0.014) (Supplementary Table 2).

**TABLE 3:** Results of meta-regression analyses on the association between plaza dancing and psychological well-being and ill-being.

	β	95% CI	*P*
**(A) Psychological well-being (*n* = 56 analyses)**			
Publication year	0.103	−0.003, 0.210	0.058
Total sample size	0.004	0.002, 0.007	**<0.001**
Mean age	–0.030	−0.070, 0.011	0.150
Duration of plaza dancing (weeks)	–0.044	−0.085, −0.004	**0.033**
Frequency of plaza dancing (times/week)	0.122	0.024, 0.221	**0.016**
Length of plaza dancing (mins/time)	0.046	−0.021, 0.113	0.175
**(B) Psychological ill**−**being (*n* = 39 analyses)**			
Publication year	0.078	−0.009, 0.166	0.079
Total sample size	–0.004	−0.013, 0.005	0.393
Mean age	–0.032	−0.109, 0.044	0.396
Duration of plaza dancing (weeks)	–0.029	−0.040, −0.018	**<0.001**
Frequency of plaza dancing (times/week)	0.154	0.030, 0.278	**0.016**
Length of plaza dancing (mins/time)	0.098	−0.022, 0.217	0.106

*Seven studies reported the results of psychological well-being, three studies reported the results of psychological ill-being, and seven studies reported the results of both psychological well-being and ill-being. Results from 14 studies (reported 56 analyses, including different related outcomes) in psychological well-being and 10 studies (reported 39 analyses, including different related outcomes) in psychological ill-being were included, respectively.*

*Meta-regression analysis was used to evaluate the heterogeneity of different studies, adjusting for publication year, total sample size, mean age, duration, frequency, and length of plaza dancing method. Numbers in bold indicate significance.*

### Assessment of Publication Bias

There was no indication of publication bias in the psychological well-being and ill-being studies, as indicated by funnel plots (Supplementary Figure 2). Egger’s and Begg’s tests yielded results similar to the funnel plots: psychological well-being (Egger *P* = 0.075; Begg *P* = 0.227) and psychological ill-being (Egger *P* = 0.304; Begg *P* = 0.056) (Supplementary Table 3).

## Discussion

To our knowledge, this is the first systematic review and meta-analysis to summarize the evidence regarding the effect of plaza dancing on psychological well-being and psychological ill-being. Overall, this study shows that plaza dancing is a useful strategy to foster the development of psychological well-being (i.e., life satisfaction, energy, happiness, positive affect, general well-being, self-image, quality of life, social functioning, and role-emotional functioning) and reduce psychological ill-being (i.e., negative affect, somatization, depression, anxiety, hostility, terror, paranoia, psychosis, nervousness, fatigue, panic, and TMD). In addition, the effect of plaza dancing on psychological well-being and ill-being is moderated by intervention modality.

Previous reviews have reported that the effects of dance intervention could improve fitness and mental health (Predovan et al., 2018; Barnish and Barran, 2020; Wu et al., 2021). However, these reviews focused only on dance intervention (e.g., ballet and folk dance) and did not pay attention to plaza dancing. The available evidence has indicated that plaza dancing may have a favorable effect on psychological well-being and ill-being (Gao et al., 2016; Wang et al., 2020). As a kind of aerobic exercise, plaza dancing is regarded not only as a form of physical activity but also as a form of mental activity and social interaction (Shen, 2016). Plaza dancing has a strong sense of musical rhythm and a cheerful melody, and the body produces neurotransmitter chemicals that reduce depression, anxiety, and other negative emotions during exercise, which can further promote the production of positive emotions such as happiness. After plaza dancing, the participants can get together to chat about the experiences and interesting things in daily life and to vent their dissatisfaction and helplessness. This reduces the mental pressure in life, offsets negative emotions, and achieves the effect of improving the state of mind (Shen, 2016). Our findings summarize the evidence regarding the effect of plaza dancing on psychological well-being and psychological ill-being, proving that plaza dancing can fulfill the purpose of regulating the participant’s state of mind.

Our study confirmed that plaza dancing has a more positive effect on psychological well-being and ill-being than walking exercise, table tennis, running, and maintaining one’s usual lifestyle. Solitary exercises, such as walking and running, with the exercise time continuing due to a lack of necessary interpersonal communication, will lead to a sense of loneliness and reduce interest in the practice. However, plaza dancing is performed collectively with the accompaniment of upbeat background music. It can effectively eliminate the loneliness of the participant and has the effect of fully mobilizing enthusiasm for the practice (Sun et al., 2018). With advancing age, due to physiological or environmental factors, people tend to have psychological disorders such as self-abasement, depression, or anxiety. Relevant studies have reported that more attention should be given to such psychological changes in people (Qiao, 2017; Xu et al., 2020). Plaza dancing can provide people with opportunities that enable them to return to society, integrate into communities, and embody themselves as quickly as possible. Furthermore, communication or skill exchanges between the plaza dancers can help reduce loneliness and increase the sense of belonging, which will improve mood and encourage optimistic and positive mental health. Moreover, the physical process of plaza dancing is complemented by rapid changes in movement and coordination between the limbs. It not only can fully mobilize the body to involve in the activities of each joint but also contributes to core muscle stimulation. These exercise effects are crucial to maintaining a stable state of mental health (Jia, 2017; Xu et al., 2020).

In our study, most of the articles originated in China, which indicates that plaza dancing has not been given attention abroad. The evidence supports several psychosocial mechanisms that explain the effect of plaza dancing on psychological well-being and ill-being. However, there is little understanding as to whether these mechanisms play the same role in populations outside of China. Therefore, future plaza dancing studies could be extended to foreign countries. Li (2017)’s study found that the average scores of GWB and PSPP in the intervention group were much higher than those obtained in the control group, indicating that plaza dancing had a certain effect on the improvement of psychological well-being, and Zeng et al. (2020)’s study found that the average scores of SCL-90 in the intervention group were much lower than those obtained in the control group, indicating that plaza dancing had a certain effect on the treatment of depression and anxiety, which were similar to the results of our study. It seems that the study participants’ age and district may not affect the effectiveness of the plaza dancing intervention, which may be related to the fact that plaza dancing is a form of entertainment and is not restricted to age and district. These results were confirmed by previous original studies (Zhang et al., 2014; Gao et al., 2016).

Most existing studies indicated that moderate-intensity exercise can promote the mental health of participants and that the duration of the exercise should be between 20 and 30 min, which is the exercise time recommended to obtain greater psychological benefits (Yang et al., 2014). Our study found that plaza dancing duration and frequency were moderators for psychological well-being and ill-being. In our review, the duration of plaza dancing was negatively correlated with the effect, which was similar to Yang et al. (2014)’s study results and may be related to longer durations contributing to more fatigue in the body. An increase in the frequency of plaza dancing can promote a positive emotional state, reduce psychological annoyance and fatigue, and reduce negative mood (Chekroud et al., 2018; Wang et al., 2019).

Our study has several strengths. First, this is the first study to provide quantitative evidence of the relationship between plaza dancing and psychological well-being and psychological ill-being. Consequently, it provides the most comprehensive evidence that experiencing plaza dancing leads to improved psychological well-being and reduced psychological ill-being. Second, this review includes RCTs, cluster-randomized trials, and quasi-RCTs or intervention studies designed to include a control group. Third, this study takes into consideration the role of various key moderators, such as measurement instruments, outcome classification, mean age, district, and plaza dancing length, duration, and frequency. Nevertheless, some limitations should also be noted. First, 90% of the included study participants were women, and only a few were men. Clinical and public health practitioners, researchers, and policymakers need to pay attention to the psychological changes of men and encourage them to perform plaza dancing exercises. Second, the included studies were from China, and the effects of plaza dancing have not been studied abroad. Thus, our findings can only be generalized to China, and they are temporarily not applicable to foreign countries with different economic statuses and cultural backgrounds. Third, publication bias is possible, as we used *P* < 0.05 and not *P* < 0.10, and significant heterogeneity would require cautious interpretations of the results. Fourth, the mediation mechanisms for successful interventions have not been identified.

## Conclusion

Evidence from the results of the meta-analysis and the original studies demonstrates that plaza dancing has a significant positive effect on psychological well-being and psychological ill-being. Plaza dancing is a promising way to promote the development of psychological well-being and treat psychological ill-being, which can improve mood, maintain happiness and positive well-being, and reduce negative affect, depression, and anxiety. Public health efforts should focus on psychological well-being and psychological ill-being and promote plaza dancing, regardless of age and region. Future research studies should aim to conduct plaza dancing interventions in different countries. Future studies should target clinical patients as participants.

## Data Availability Statement

The original contributions presented in the study are included in the article/Supplementary Material, further inquiries can be directed to the corresponding author.

## Author Contributions

ZB, RB, and YL designed the research. YY, CX, and ZZ conducted the literature search, data screening, and extraction. YL performed the meta-analysis and drafted the original manuscript. All authors revised the manuscript, critically helped in the interpretation of the results, and provided relevant intellectual inputs. RB and YL provided administrative support for the project and hold primary responsibility for the final manuscript. All authors read and approved the final manuscript.

## Conflict of Interest

The authors declare that the research was conducted in the absence of any commercial or financial relationships that could be construed as a potential conflict of interest.

## Publisher’s Note

All claims expressed in this article are solely those of the authors and do not necessarily represent those of their affiliated organizations, or those of the publisher, the editors and the reviewers. Any product that may be evaluated in this article, or claim that may be made by its manufacturer, is not guaranteed or endorsed by the publisher.

## Footnotes

1
https://www.nhlbi.nih.gov/health-topics/study-quality-assessment-tools
