# Supplementary material for: The Effectiveness of Plaza Dancing on Psychological Well-Being and Ill-Being: A Systematic Review and Meta-Analysis
Source: Front Psychol. 2022 Apr 15;13:864327. doi: 10.3389/fpsyg.2022.864327 (PMC9051395; doi:10.3389/fpsyg.2022.864327)
Supplement: Supplementary file 1 [file Table_1.docx]

Supplementary Material

# Supplementary Text

Search strategies of the associations between plaza dancing and psychological well-being and ill-being.

1) English:

(square danc*[Title/Abstract]  OR square fitness danc*[Title/Abstract] ) AND (mental health[Title/Abstract] OR mental hygiene[Title/Abstract] OR self concept[Title/Abstract] OR self-perception[Title/Abstract] OR self-esteem[Title/Abstract] OR self efficacy[Title/Abstract] OR body image*[Title/Abstract] OR body representation*[Title/Abstract] OR body schema[Title/Abstract] OR happiness[Title/Abstract] OR affect[Title/Abstract] OR optimism[Title/Abstract] OR anxiety[Title/Abstract] OR depression[Title/Abstract] OR pessimism[Title/Abstract] OR positive affect*[Title/Abstract] OR negative affect*[Title/Abstract] OR mood*[Title/Abstract] OR hypervigilance[Title/Abstract] OR nervousness[Title/Abstract] OR depressive symptom*[Title/Abstract] OR emotional depression*[Title/Abstract] OR psychological stress*[Title/Abstract] OR life stress* [Title/Abstract]

2) Chinese:

( 广场舞) AND (心理健康[Title/Abstract] OR心理卫生[Title/Abstract] OR 自我认知[Title/Abstract] OR身体形象 [Title/Abstract] OR幸福 [Title/Abstract] OR乐观 [Title/Abstract] OR 悲观[Title/Abstract] OR抑郁[Title/Abstract] OR焦虑 [Title/Abstract] OR积极影响 [Title/Abstract] OR消极影响[Title/Abstract] OR情绪 [Title/Abstract] OR紧张[Title/Abstract] OR压抑 [Title/Abstract] OR生活压力 [Title/Abstract] OR心理压力 [Title/Abstract]

# Supplementary Figures and Tables

## Supplementary Figures


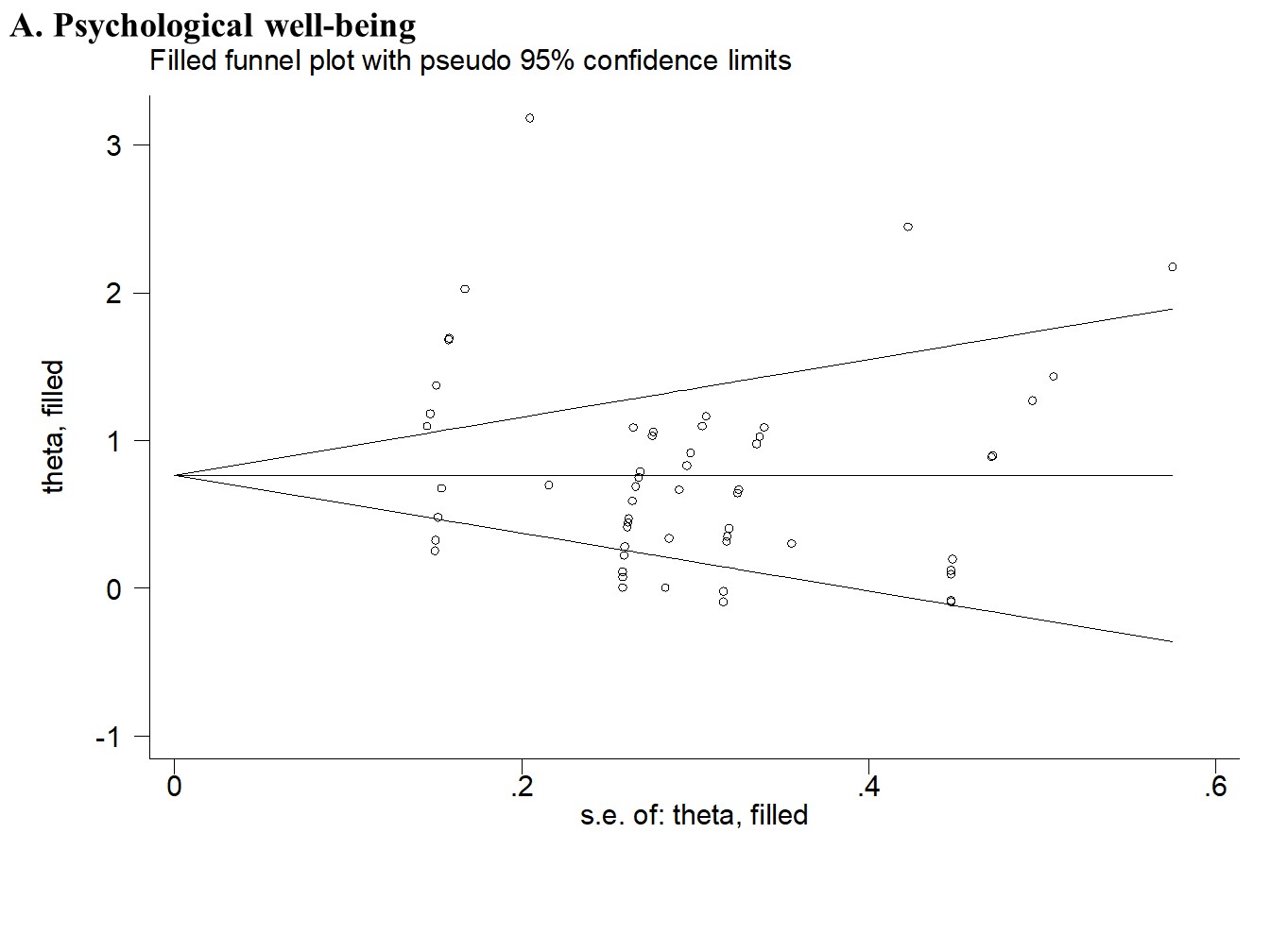

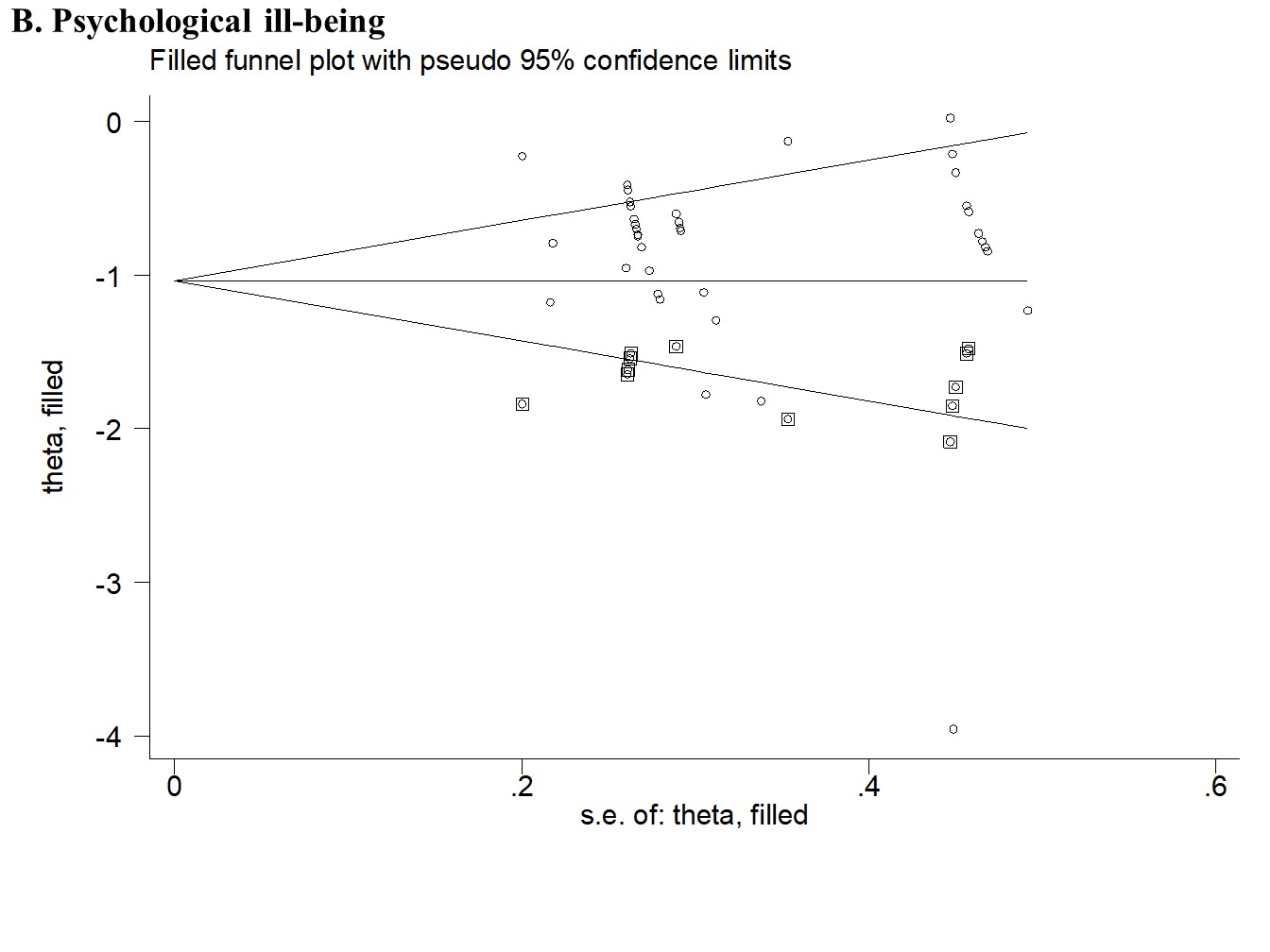


**Supplementary Figure 1.** Funnel plot for publication bias. Seven studies reported the results of psychological well-being, three studies reported the results of psychological ill-being, and seven studies reported the results of both psychological well-being and ill-being. Results from fourteen studies (reported 56 analyses, including for different related outcomes) in psychological well-being, and ten studies (reported 39 analyses, including for different related outcomes) in psychological ill-being were included, respectively. We used the trim method to make the funnel plot.

## Supplementary Tables

**Supplementary Table 1.** Study quality assessment for the included studies

| **Study ID/ NIH Criteria** | 1 | 2 | 3 | 4 | 5 | 6 | 7 | 8 | 9 | 10 | 11 | 12 | 13 | 14 | 15 | 16 | 17 | 18 | 19 | 20 | 21 | 22 | 23 | 24 | 25 |
| --- | --- | --- | --- | --- | --- | --- | --- | --- | --- | --- | --- | --- | --- | --- | --- | --- | --- | --- | --- | --- | --- | --- | --- | --- | --- |
| 1 | Y | N | N | N | N | N | Y | N | Y | Y | N | Y | Y | N | N | Y | N | N | N | Y | Y | Y | Y | Y | N |
| 2 | Y | N | N | N | N | N | Y | N | Y | Y | N | Y | Y | N | N | Y | N | N | N | Y | Y | Y | Y | Y | N |
| 3 | NR | N | N | N | N | N | Y | N | NR | Y | N | NR | NR | N | N | NR | N | N | N | NR | NR | NR | NR | NR | N |
| 4 | NR | N | N | N | N | N | N | N | NR | N | N | NR | NR | N | N | NR | N | N | N | NR | NR | NR | NR | NR | N |
| 5 | NR | N | N | N | N | N | Y | N | NR | NR | N | NR | NR | N | N | NR | N | N | N | NR | NR | NR | NR | NR | N |
| 6 | Y | Y | Y | Y | Y | Y | Y | Y | Y | Y | Y | Y | Y | Y | Y | Y | Y | Y | Y | NR | Y | Y | NR | Y | Y |
| 7 | Y | Y | Y | Y | Y | Y | Y | Y | Y | Y | Y | Y | Y | Y | Y | Y | Y | Y | Y | Y | Y | Y | Y | Y | Y |
| 8 | Y | Y | Y | Y | Y | Y | Y | Y | Y | Y | Y | Y | Y | Y | Y | Y | Y | Y | Y | Y | Y | Y | Y | Y | Y |
| 9 | Y | NR | NR | NR | NR | Y | Y | NR | NR | NR | NR | Y | NR | NR | NR | NR | Y | NR | NR | Y | Y | Y | NR | Y | Y |
| 10 | Y | Y | Y | Y | Y | Y | Y | Y | Y | Y | Y | Y | Y | Y | Y | Y | Y | Y | Y | Y | Y | Y | Y | Y | Y |
| 11 | Y | Y | Y | Y | Y | Y | Y | Y | Y | Y | Y | Y | Y | Y | Y | Y | Y | Y | Y | Y | Y | Y | Y | Y | Y |
| 12 | N | N | N | N | N | Y | N | N | N | N | N | N | N | N | N | Y | N | N | N | Y | Y | Y | N | N | Y |
| 13 | Y | Y | Y | Y | Y | Y | Y | Y | Y | Y | Y | Y | Y | Y | Y | Y | Y | Y | Y | Y | Y | Y | Y | Y | Y |
| 14 | N | Y | Y | Y | Y | Y | Y | Y | Y | Y | Y | Y | Y | Y | Y | Y | Y | Y | Y | N | Y | Y | Y | Y | Y |
| Total score | 9 | 7 | 7 | 7 | 7 | 9 | 12 | 7 | 9 | 10 | 7 | 10 | 9 | 7 | 7 | 10 | 8 | 7 | 7 | 9 | 11 | 11 | 8 | 10 | 9 |
| Mean score | 8.56 | | | | | | | | | | | | | | | | | | | | | | | | |

Abbreviations: NR, Not Report; Y, Yes; N, No. This study quality assessment tool was adopted from the National Institutes of Health’s Quality Assessment Tool for Controlled Intervention Studies. For each criterion, a score of one was assigned if “Y” was the response, whereas a score of zero was assigned otherwise (i.e., an answer of “no”, “not applicable”, “not reported”, or “cannot determine”). A study-specific global score, ranging from zero to 14, was calculated by summing up scores across all 14 criteria.

**Supplementary Table 2.** Sensitivity analysis on the associations between plaza dancing and psychological well-being and ill-being

| Study omitted | Instruments | Outcome | Effect Size (95% CI) | I^2^ (%) | χ^2^ | P value |
| --- | --- | --- | --- | --- | --- | --- |
| **A. Psychological well-being** | | | | | | |
| Yongsheng Wang (2021) | LSIA | Life satisfaction | 0.77 (0.58, 0.95) | 87.1 | 419.00 | <0.001 |
| Yi Yu (2021) | GWB | Energy | 0.77 (0.58, 0.95) | 87.1 | 419.15 | <0.001 |
| Yi Yu (2021) | GWB | Life satisfaction | 0.74 (0.55, 0.92) | 86.7 | 405.58 | <0.001 |
| Yi Yu (2021) | GWB | Happiness | 0.77 (0.58, 0.96) | 87.0 | 416.61 | <0.001 |
| Yi Yu (2021) | GWB | Positive affect | 0.76 (0.57, 0.95) | 87.1 | 419.41 | <0.001 |
| Yi Yu (2021) | GWB | Relaxation | 0.78 (0.59, 0.96) | 86.9 | 411.64 | <0.001 |
| Jun Zeng (2020) | POMS | Energy | 0.77 (0.58, 0.96) | 87.1 | 418.53 | <0.001 |
| Jun Zeng (2020) | POMS | Self-esteem | 0.78 (0.59, 0.96) | 86.8 | 408.20 | <0.001 |
| Meize Sun (2019) | GWB | Energy | 0.76 (0.57, 0.95) | 87.1 | 417.01 | <0.001 |
| Meize Sun (2019) | GWB | Life satisfaction | 0.76 (0.56, 0.95) | 87.0 | 414.83 | <0.001 |
| Meize Sun (2019) | GWB | Happiness | 0.75 (0.56, 0.94) | 86.8 | 408.02 | <0.001 |
| Meize Sun (2019) | GWB | Positive affect | 0.74 (0.56, 0.93) | 86.2 | 391.24 | <0.001 |
| Meize Sun (2019) | GWB | Relaxation | 0.74 (0.56, 0.92) | 85.4 | 369.72 | <0.001 |
| Meize Sun (2019) | GWB | General well-being | 0.72 (0.56, 0.88) | 81.3 | 288.41 | <0.001 |
| Meize Sun (2019) | PSPP | Self-image | 0.74 (0.56, 0.93) | 86.2 | 391.92 | <0.001 |
| Shou Wang (2019) | SF-36 | Quality of life | 0.76 (0.57, 0.95) | 87.1 | 418.94 | <0.001 |
| Xiaolin Wu (2019) | GWB | Energy | 0.76 (0.57, 0.95) | 87.1 | 419.62 | <0.001 |
| Xiaolin Wu (2019) | GWB | Life satisfaction | 0.76 (0.57, 0.95) | 87.1 | 419.05 | <0.001 |
| Xiaolin Wu (2019) | GWB | Happiness | 0.76 (0.57, 0.95) | 87.1 | 419.61 | <0.001 |
| Xiaolin Wu (2019) | GWB | Positive affect | 0.78 (0.59, 0.96) | 86.8 | 410.13 | <0.001 |
| Xiaolin Wu (2019) | GWB | Relaxation | 0.77 (0.58, 0.96) | 87.0 | 416.15 | <0.001 |
| Qingfang Guo (2017) | SES | Self-esteem | 0.77 (0.58, 0.96) | 87.1 | 417.06 | <0.001 |
| Shiqi Li (2017) | PSPP | Self-image | 0.76 (0.58, 0.95) | 87.1 | 419.45 | <0.001 |
| Shiqi Li (2017) | GWB | Energy | 0.78 (0.59, 0.96) | 86.9 | 410.92 | <0.001 |
| Shiqi Li (2017) | GWB | Life satisfaction | 0.76 (0.57, 0.95) | 87.1 | 419.15 | <0.001 |
| Shiqi Li (2017) | GWB | Happiness | 0.77 (0.59, 0.96) | 86.9 | 413.32 | <0.001 |
| Shiqi Li (2017) | GWB | Positive affect | 0.77 (0.58, 0.96) | 87.0 | 416.98 | <0.001 |
| Shiqi Li (2017) | GWB | Relaxation | 0.76 (0.57, 0.95) | 87.1 | 419.29 | <0.001 |
| Ying Pang (2017) | PSPP | Self-image | 0.76 (0.57, 0.95) | 87.1 | 419.21 | <0.001 |
| Ying Pang (2017) | GWB | Life satisfaction | 0.76 (0.57, 0.95) | 87.1 | 419.52 | <0.001 |
| Ying Pang (2017) | GWB | Energy | 0.77 (0.58, 0.96) | 87.1 | 417.50 | <0.001 |
| Ying Pang (2017) | GWB | Happiness | 0.77 (0.58, 0.96) | 87.1 | 417.01 | <0.001 |
| Ying Pang (2017) | GWB | Positive affect | 0.78 (0.59, 0.97) | 86.8 | 410.37 | <0.001 |
| Ying Pang (2017) | GWB | Relaxation | 0.77 (0.58, 0.95) | 87.1 | 419.25 | <0.001 |
| Wei Wang (2017) | GWB | Energy | 0.78 (0.59, 0.96) | 86.8 | 410.06 | <0.001 |
| Wei Wang (2017) | GWB | Life satisfaction | 0.77 (0.58, 0.96) | 87.1 | 417.34 | <0.001 |
| Wei Wang (2017) | GWB | Happiness | 0.77 (0.58, 0.95) | 87.1 | 419.18 | <0.001 |
| Wei Wang (2017) | GWB | Positive affect | 0.77 (0.58, 0.96) | 87.0 | 416.55 | <0.001 |
| Wei Wang (2017) | GWB | Relaxation | 0.77 (0.59, 0.96) | 87.0 | 414.47 | <0.001 |
| Wei Wang (2017) | GWB | General well-being | 0.76 (0.58, 0.95) | 87.1 | 419.54 | <0.001 |
| Lili Han (2016) | POMS | Energy | 0.76 (0.57, 0.94) | 87.1 | 418.69 | <0.001 |
| Lili Han (2016) | POMS | Self-esteem | 0.77 (0.58, 0.95) | 87.1 | 419.15 | <0.001 |
| Jiayan Li (2016) | EIEQ | Energetic stimulation | 0.78 (0.59, 0.96) | 87.0 | 415.11 | <0.001 |
| Jiayan Li (2016) | EIEQ | Calm mind and body | 0.78 (0.59, 0.96) | 87.0 | 415.00 | <0.001 |
| Jiayan Li (2016) | EIEQ | Active involvement | 0.77 (0.59, 0.96) | 87.0 | 416.61 | <0.001 |
| Jiayan Li (2016) | CSS | Self-concept | 0.77 (0.59, 0.96) | 87.1 | 417.38 | <0.001 |
| Lin Shen (2016) | GWB | Life satisfaction | 0.76 (0.57, 0.94) | 87.1 | 418.39 | <0.001 |
| Lin Shen (2016) | GWB | Energy | 0.76 (0.58, 0.95) | 87.1 | 419.63 | <0.001 |
| Lin Shen (2016) | GWB | Happiness | 0.76 (0.57, 0.94) | 87.1 | 418.96 | <0.001 |
| Lin Shen (2016) | GWB | Positive affect | 0.76 (0.58, 0.95) | 87.1 | 419.63 | <0.001 |
| Lin Shen (2016) | GWB | Relaxation | 0.77 (0.59, 0.96) | 87.0 | 416.83 | <0.001 |
| Lin Shen (2016) | GWB | General well-being | 0.75 (0.56, 0.93) | 87.0 | 414.44 | <0.001 |
| Hongyu Wang (2016) | SF-36 | Vitality | 0.78 (0.59, 0.96) | 86.6 | 401.97 | <0.001 |
| Hongyu Wang (2016) | SF-36 | Social functioning | 0.77 (0.58, 0.96) | 86.9 | 412.87 | <0.001 |
| Hongyu Wang (2016) | SF-36 | Role-emotional | 0.77 (0.58, 0.96) | 87.1 | 418.03 | <0.001 |
| Hongyu Wang (2016) | SF-36 | Mental health | 0.77 (0.59, 0.96) | 86.7 | 406.10 | <0.001 |
| **B. Psychological ill-being** | | | | | | |
| Yongsheng Wang (2021) | PEES | Negative affect | -0.84 (-1.01, -0.68) | 65.7 | 107.96 | <0.001 |
| Jun Zeng (2020) | SCL-90 | Somatization | -0.84 (-1.01, -0.68) | 65.7 | 107.90 | <0.001 |
| Jun Zeng (2020) | SCL-90 | Compulsive symptoms | -0.85 (-1.02, -0.69) | 65.1 | 105.90 | <0.001 |
| Jun Zeng (2020) | SCL-90 | Interpersonal sensitivity | -0.84 (-1.01, -0.67) | 65.6 | 107.63 | <0.001 |
| Jun Zeng (2020) | SCL-90 | Depression | -0.85 (-1.01, -0.68) | 65.6 | 107.65 | <0.001 |
| Jun Zeng (2020) | SCL-90 | Anxiety | -0.85 (-1.01, -0.68) | 65.7 | 107.77 | <0.001 |
| Jun Zeng (2020) | SCL-90 | Hostility | -0.83 (-1.00, -0.67) | 65.3 | 106.73 | <0.001 |
| Jun Zeng (2020) | SCL-90 | Terror | -0.83 (-1.00, -0.67) | 65.2 | 106.41 | <0.001 |
| Jun Zeng (2020) | SCL-90 | Paranoid | -0.83 (-1.00, -0.67) | 65.2 | 106.40 | <0.001 |
| Jun Zeng (2020) | SCL-90 | Psychosis | -0.85 (-1.02, -0.68) | 65.6 | 107.50 | <0.001 |
| Jun Zeng (2020) | POMS | Nervousness | -0.85 (-1.01, -0.68) | 65.7 | 107.88 | <0.001 |
| Jun Zeng (2020) | POMS | Anger | -0.85 (-1.02, -0.69) | 65.3 | 106.67 | <0.001 |
| Jun Zeng (2020) | POMS | Fatigue | -0.85 (-1.01, -0.68) | 65.7 | 107.88 | <0.001 |
| Jun Zeng (2020) | POMS | Panic | -0.85 (-1.02, -0.68) | 65.4 | 106.95 | <0.001 |
| Jun Zeng (2020) | POMS | Depression | -0.85 (-1.02, -0.69) | 64.9 | 105.50 | <0.001 |
| Jun Zeng (2020) | POMS | TMD | -0.84 (-1.01, -0.68) | 65.7 | 107.97 | <0.001 |
| Shou Wang (2019) | GDS-15 | Depression | -0.84 (-1.01, -0.67) | 65.6 | 107.68 | <0.001 |
| Qingfang Guo (2017) | BDI | Depression | -0.86 (-1.02, -0.70) | 64.5 | 104.09 | <0.001 |
| Ziyao Guan (2017) | SAS | Anxiety | -0.83 (-1.00, -0.66) | 64.8 | 105.01 | <0.001 |
| Ziyao Guan (2017) | SDS | Depression | -0.86 (-1.02, -0.70) | 62.5 | 98.77 | <0.001 |
| Lei Gao (2016) | SDS | Depression | -0.83 (-0.99, -0.66) | 64.9 | 105.55 | <0.001 |
| Lili Han (2016) | POMS | Nervousness | -0.85 (-1.01, -0.68) | 65.7 | 107.80 | <0.001 |
| Lili Han (2016) | POMS | Anger | -0.82 (-0.98, -0.66) | 62.6 | 98.99 | <0.001 |
| Lili Han (2016) | POMS | Fatigue | -0.85 (-1.01, -0.68) | 65.6 | 107.65 | <0.001 |
| Lili Han (2016) | POMS | Depression | -0.85 (-1.02, -0.68) | 65.5 | 107.39 | <0.001 |
| Lili Han (2016) | POMS | Panic | -0.85 (-1.01, -0.68) | 65.7 | 107.85 | <0.001 |
| Lili Han (2016) | POMS | TMD | -0.83 (-1.00, -0.67) | 65.4 | 106.98 | <0.001 |
| Jiayan Li (2016) | EIEQ | Physical exhaustion | -0.85 (-1.02, -0.69) | 65.1 | 106.13 | <0.001 |
| Lin Shen (2016) | SCL-90 | Somatization | -0.84 (-1.01, -0.68) | 65.7 | 107.93 | <0.001 |
| Lin Shen (2016) | SCL-90 | Compulsive symptoms | -0.85 (-1.02, -0.69) | 65.4 | 106.81 | <0.001 |
| Lin Shen (2016) | SCL-90 | Interpersonal sensitivity | -0.86 (-1.02, -0.70) | 64.6 | 104.40 | <0.001 |
| Lin Shen (2016) | SCL-90 | Depression | -0.85 (-1.01, -0.68) | 65.6 | 107.63 | <0.001 |
| Lin Shen (2016) | SCL-90 | Anxiety | -0.84 (-1.01, -0.68) | 65.7 | 107.97 | <0.001 |
| Lin Shen (2016) | SCL-90 | Hostility | -0.84 (-1.01, -0.68) | 65.7 | 107.97 | <0.001 |
| Lin Shen (2016) | SCL-90 | Terror | -0.84 (-1.01, -0.68) | 65.7 | 107.97 | <0.001 |
| Lin Shen (2016) | SCL-90 | Paranoid | -0.85 (-1.01, -0.68) | 65.7 | 107.72 | <0.001 |
| Lin Shen (2016) | SCL-90 | Psychosis | -0.84 (-1.00, -0.67) | 65.5 | 107.25 | <0.001 |
| Xinan Zhang (2014) | HAMA | Anxiety | -0.78 (-0.91, -0.66) | 36.7 | 58.48 | 0.014 |
| Xinan Zhang (2014) | HAMD | Depression | -0.82 (-0.97, -0.66) | 62.2 | 97.88 | <0.001 |

**Note:** Seven studies reported the results of psychological well-being, three studies reported the results of psychological ill-being, and seven studies reported the results of both psychological well-being and ill-being. Results from fourteen studies (reported 56 analyses, including for different related outcomes) in psychological well-being, and ten studies (reported 39 analyses, including for different related outcomes) in psychological ill-being were included, respectively.

Abbreviation: BDI, Beck depression rating scale; CSS, Core Self-evaluation Scale; EIEQ, Exercise Induced Emotion Questionnaire; GDS-15, 15-item Geriatric Depression Scale; GWB, General Well-Being Schedule; HAMA, Hamilton Anxiety Scale; HAMD, Hamilton Depression Scale; LSIA, Life Satisfaction Index A; PEES, Post-exercise Emotional Experience Scale; POMS, Profile of Mood States; PSPP, Platform Sizing and Performance Program; SAS, Self-Rating Anxiety Scale; SCL-90, Symptom Check List 90; SDS, Self-Rating Depression Scale; SES, Self-esteem scale; SF-36, the MOS item short from health survey; TMD, Total of motional disturb.

**Supplementary Table 3.** The Egger’s and Begg’s test for publication bias of the included studies

|  | Psychological well-being | Psychological ill-being |
| --- | --- | --- |
| P (Egger’s test) | 0.075 | 0.304 |
| P (Begg’s test) | 0.227 | 0.056 |

**Note:** Seven studies reported the results of psychological well-being, three studies reported the results of psychological ill-being, and seven studies reported the results of both psychological well-being and ill-being. Results from fourteen studies (reported 56 analyses, including for different related outcomes) in psychological well-being, and ten studies (reported 39 analyses, including for different related outcomes) in psychological ill-being were included, respectively.
